# Supplementary material for: Cytomegalovirus-Specific Hyperimmune Immunoglobulin Administration for Secondary Prevention after First-Trimester Maternal Primary Infection: A 13-Year Single-Center Cohort Study
Source: Viruses. 2024 Aug 2;16(8):1241. doi: 10.3390/v16081241 (PMC11359551; doi:10.3390/v16081241)

Table S1. Association of transmission outcome with baseline characteristics of women starting HIG treatment in the first trimester (≤14th GW).

	**Transmission (-), n=51**	**Transmission (+), n=20**	***p value***
Age, mean (SD)	30.6 (4.6)	33 (4.9)	0.06
Gestational week at diagnosis, median (interquartile range)	8 (3)	8 (3)	0.92
Gestational week at first HIG dose, median (interquartile range)	11 (3)	11 (3)	0.531
Time from diagnosis to first HIG dose (weeks), median (interquartile range)	2 (3)	2 (2)	0.387
Time from diagnosis to first HIG dose, n (%)			
≤2 weeks	30 (58.8)	14/19 (73.7)	0.253
>2 weeks	21 (41.2)	5/19 (26.3)	
Time from diagnosis to first HIG dose, n (%)			
≤4 weeks	38 (74.5)	18/19 (94.7)	0.06
>4 weeks	13 (25.5)	1/19 (5.3)	
Number of HIG doses, median (interquartile range)	5 (2)	5.5 (3)	0.318
Maternal viremia, *n/N* (%)	12/37 (32.4)	6/12 (50)	0.273

GW: gestational week, HIG: hyperimmune globulin, n: number, SD: standard deviation

Table S2. Baseline characteristics of women starting HIG treatment during the first (≤14th GW) vs. second trimester (>15th GW)

	**Start of HIG treatment in the first trimester (n=71)**	**Start of HIG treatment in the second trimester (n=36)**	***p value***
Age, mean (SD)	31.3 (4.7)	32.2 (5.3)	0.354
Gestation week at diagnosis, median (interquartile range)	8 (7-10)	11 (9-13)	<0.0001
Gestation week at first HIG dose, median (interquartile range)	11 (9-13)	16 (15-17)	<0.0001
Time from diagnosis to start of HIG treatment (weeks), median (interquartile range)	2 (1-3)	5 (3-7)	<0.0001
Number of HIG doses, median (interquartile range)	5 (4-7)	3.5 (3-5)	<0.0001
Maternal viremia, *n*/*N* (%)	18/49 (36.7)	9/28 (32.1)	0.685
Transmission rate, *n* (%)	20 (28.2)	12 (33.3)	0.58

GW: gestational week, HIG: hyperimmune globulin, SD: standard deviation

Figure S1. Total cohort flowchart. CMV: cytomegalovirus, IgG: immunoglobulin G, IgM: immunoglobulin M, n: num

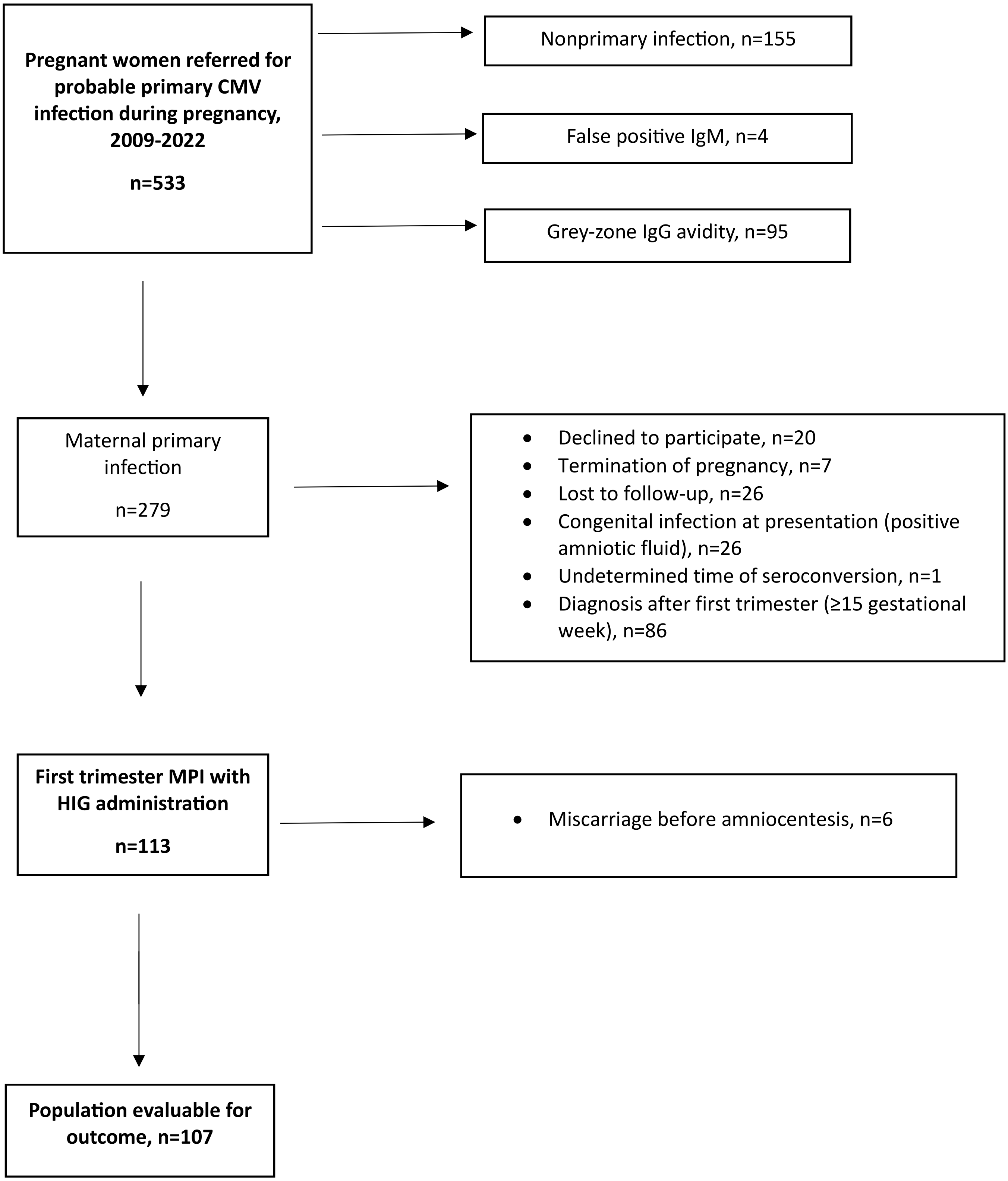

Supplement: Supplementary file 1 [file viruses-16-01241-s001.zip › viruses-3118683-supplementary.pdf]
